# Environmental drivers of spatial patterns of topsoil nitrogen and phosphorus under monsoon conditions in a complex terrain of South Korea

**DOI:** 10.1371/journal.pone.0183205

**Published:** 2017-08-24

**Authors:** Gwanyong Jeong, Kwanghun Choi, Marie Spohn, Soo Jin Park, Bernd Huwe, Mareike Ließ

**Affiliations:** 1 Department of Geosciences/ Soil Physics Division, Bayreuth Center of Ecology and Environmental Research (BayCEER), University of Bayreuth, Bayreuth, Germany; 2 Biogeographical Modelling, Bayreuth Center of Ecology and Environmental Research (BayCEER), University of Bayreuth, Bayreuth, Germany; 3 Department of Soil Ecology, Bayreuth Center of Ecology and Environmental Research (BayCEER), University of Bayreuth, Bayreuth, Germany; 4 Department of Geography, Seoul National University, Shilim-dong, Kwanak-gu, Seoul, South Korea; 5 Department of Soil Physics, Helmholtz Centre for Environmental Research–UFZ, Halle, Germany; The University of Sydney, AUSTRALIA

## Abstract

Nitrogen (N) and phosphorus (P) in topsoils are critical for plant nutrition. Relatively little is known about the spatial patterns of N and P in the organic layer of mountainous landscapes. Therefore, the spatial distributions of N and P in both the organic layer and the A horizon were analyzed using a light detection and ranging (LiDAR) digital elevation model and vegetation metrics. The objective of the study was to analyze the effect of vegetation and topography on the spatial patterns of N and P in a small watershed covered by forest in South Korea. Soil samples were collected using the conditioned latin hypercube method. LiDAR vegetation metrics, the normalized difference vegetation index (NDVI), and terrain parameters were derived as predictors. Spatial explicit predictions of N/P ratios were obtained using a random forest with uncertainty analysis. We tested different strategies of model validation (repeated 2-fold to 20-fold and leave-one-out cross validation). Repeated 10-fold cross validation was selected for model validation due to the comparatively high accuracy and low variance of prediction. Surface curvature was the best predictor of P contents in the organic layer and in the A horizon, while LiDAR vegetation metrics and NDVI were important predictors of N in the organic layer. N/P ratios increased with surface curvature and were higher on the convex upper slope than on the concave lower slope. This was due to P enrichment of the soil on the lower slope and a more even spatial distribution of N. Our digital soil maps showed that the topsoils on the upper slopes contained relatively little P. These findings are critical for understanding N and P dynamics in mountainous ecosystems.

## Introduction

Nitrogen (N) and phosphorus (P) are the most important nutrients for primary productivity in terrestrial ecosystems [1,2]. Soil nutrient content varies during long-term soil development, such that N increases while P declines during the course of pedogenesis. This is because N enters the ecosystem via N-fixing microorganisms, whereas P is derived from the weathering of minerals. As a result, primary productivity is initially N-limited in lightly weathered soils but becomes increasingly P-limited in highly weathered soils over millions of years [3].

P limitation is enhanced by atmospheric N deposition [2,4]. In East Asia, where the population and economy are growing rapidly, atmospheric N deposition is currently very high [5]. In South Korea, atmospheric N inputs have rapidly increased due to large industrial operations and agricultural intensification [6–8]. The annual average wet input of N ranged from 12.9 to 24.9 kg ha^-1^year^-1^ from 2005 to 2010 [6], and is markedly higher than that during pre-industrial times. This might have effects on the productivity, biodiversity, and community composition of plants [9].

An understanding of nutrient contents in the organic layer is critical for mountainous ecosystem management. Organic layers are made up of freshly fallen organic matter, including whole leaves, twigs, and fruits. Following mineralization of organic matter, the organic layer slowly supplies nutrients, which are absorbed by plant roots [10]. Therefore, nutrients that are returned to soil by litterfall are important for plant nutrition [11]. In particular, the N/P ratio in topsoil is used as an indicator of potential growth limitation [12], and the spatial patterns of nutrients in the organic layer and in the A horizon can provide insight into soil-vegetation relationships.

Many studies have assessed spatial patterns of soil N [13–15] and P [16–18]. Previous studies on mountain ecosystems have found environmental correlations between the N contents in the organic layer and topographic parameters in a temperate forested watershed [19] and in boreal forests [20]. Wilcke et al. [21] reported an elevation gradient of decreasing N and P content in organic layers, and Soethe et al. [22] found that the N stocks of the organic layer differ significantly between different elevations in tropical mountain forests. However, our understanding of quantitative relationships between the content of nutrients (especially P) in the organic layer, topography, and vegetation is limited. In this regard, recent advances in digital soil mapping (DSM) have allowed us to improve our knowledge on spatial patterns of N and P and their environmental controls.

DSM often uses topographical predictors derived from digital elevation models (DEM), such as elevation, slope angle, curvature, and wetness index [23,24]. According to Ballabio [25], maps of soil properties can be produced with good accuracy using only terrain parameters as predictors in mountainous areas. In addition, vegetation data might improve DSM results, especially for the organic layer since it strongly depends on the vegetation [26]. Various vegetation parameters derived from satellite images have helped to explain the spatial variability of soil nutrients when used as DSM predictors [27,28]. However, to our knowledge, no attempt has been made to use Light detection and ranging (LiDAR) derived vegetation metrics for the spatial predictions of soil properties.

LiDAR-derived vegetation metrics could extend our understanding of spatial soil data by providing insight into the relationship between soils and vegetation as they are related to the vegetation’s vertical variability, which reflects forest structure metrics [29]. Canopy cover percentage and maximum height can indicate the above ground biomass and forest productivity [30]. LiDAR predictors may also act as ecological indicators, such as light condition on the forest floor [30]. LiDAR intensity varies with land cover and forest types [31]. Additionally, LiDAR predictors are high-resolution data, which provide more detailed spatial information than can be obtained from other types of remote sensing data (e.g. Aster [15 m] or Landsat [30 m] images). The normalized difference vegetation index (NDVI) and LiDAR data are expected to be important for N predictions related to forest biomass, but most probably not for P since it is assumed to mainly originate from bedrock.

LiDAR DEM could also be useful for predicting the spatial distributions of soil nutrients, especially P. P in soils tends to be fixed into stable forms as iron, aluminium, and calcium combinations [32]. Most P in soils is lost by soil erosion and is moved along surface configuration [33]. The LiDAR DEM can provide high resolution information on topography which might benefit the investigation of spatial P patterns.

To better understand the spatial patterns of N and P in the organic layer and mineral topsoil, the aim of this study was to use high-resolution LiDAR data and the derived DEM and vegetation metrics to predict topsoil N and P content by a DSM regression approach. The specific objectives of our research were: (1) to test the importance of LiDAR-derived vegetation and topographical parameters to understand the spatial patterns of N and P; (2) to identify subareas with critical P contents; and (3) to test different validation strategies for N and P.

## Materials and methods

### Research area

**Fig 1:**
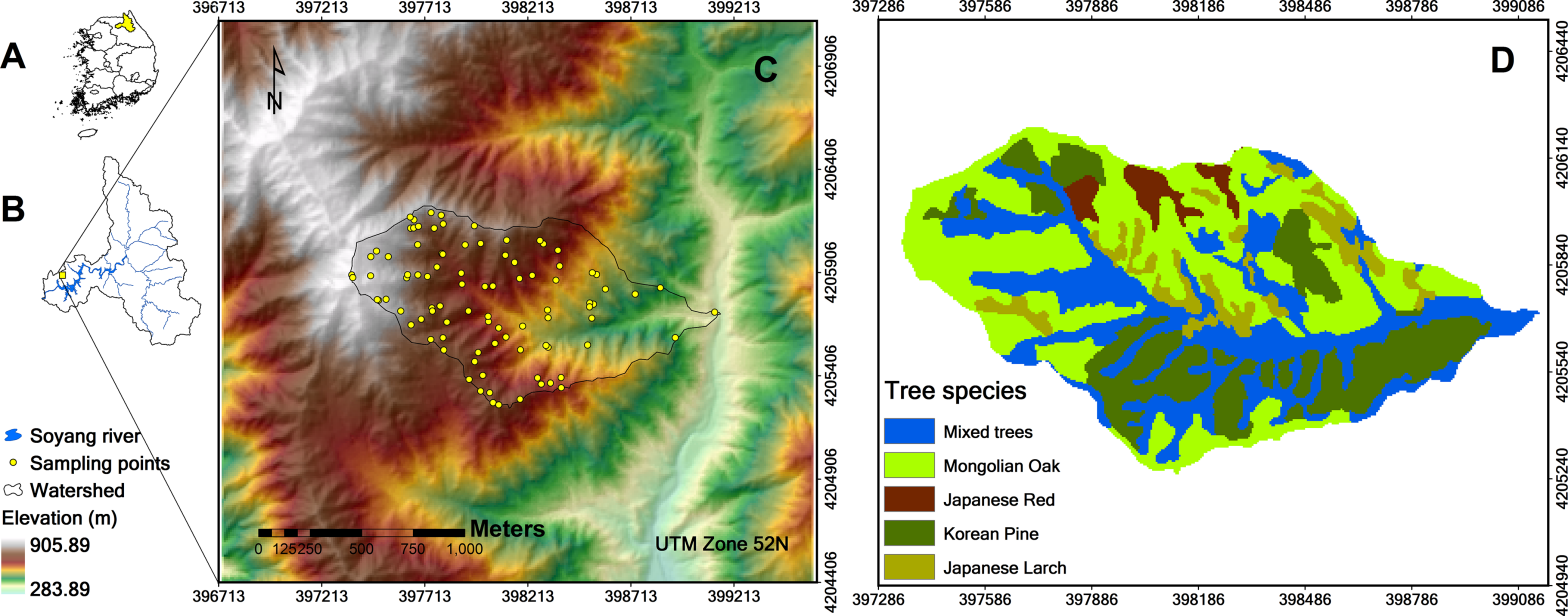
Research area. (A) The Soyang watershed within South Korea. (B) The research area within the Soyang watershed. (C) The research area with the sampling points. (D) The tree species map (fgis.forest.go.kr/).

The study area has a size of 9.84 km^2^ and is located in the downstream area of the Soyang lake watershed, Gangwon province, South Korea (Fig 1). The mean annual air temperature of the study area is 11.1°C and it receives a mean annual rainfall of 1,347 mm [34] with about 70% of the annual rain (824.4 mm) falling in the summer monsoon season (June, July, and August) [34]. The area’s bedrock is part of the Gyeonggi gneiss complex, which consists of granitic gneiss and banded gneiss [35] formed in the Paleoproterozoic and belonging to the oldest basement rocks in the Korean Peninsula [36]. The elevation ranges between 320 and 868 m above sea level and the area consists of various steep slopes (over 45°) caused by a tectonic uplift that occurred during the Quaternary Period [37]. The area is a headwater catchment with narrow depositional areas and valleys, and plays an important role in the biogeochemical cycle of the downstream hydrological system as a key source of nutrients [38]. Its soils are mainly composed of fine gravelly sandy loam soils, fine sandy loam, and gravelly loam soils [39]. The area is part of a national forest and the main tree species are Mongolian oak (*Quercus mongolica*; 40–50 years) and Korean pine (*Pinus koraiensis*; 30–35 years), locally vegetated with Japanese red pine (*Pinus densiflora*) and Japanese larch (*Larix kaempferi*) (Fig 1).

### Soil sampling and chemical analyses

Soil samples were collected from the organic layer and the A horizon at 91 sampling sites in 2014. Spatial position information of sampling points was recorded with a Qmini H3 global navigation satellite system (GNSS) GPS (accuracy within 5 m). Field studies were carried out under research permission from the Korea Forest Service of Chuncheon. We confirm that the field studies did not involve endangered or protected species. Conditioned Latin Hypercube Sampling (cLHS) was applied to optimize the density functions of the n-dimensional covariate space for the regression models [40]. This is a stratified random sampling approach that divides the empirical density functions of the predictor space into quantiles based on the number of samples. In order to obtain a Latin hypercube of exactly one sample per quantile for each of the predictors, an optimization approach is used. In the R package "clhs" [41], this is achieved by simulated annealing.

The organic layer had an average depth of 5 cm and was sampled using a metal frame of 0.3 x 0.3 m. The A horizon of the mineral soil was sampled using a shovel according to the depth of the A horizon, which differed between 10 and 30 cm. Mineral soil samples were air-dried and sieved (< 2 mm). The organic layer samples were oven-dried. Total P was extracted with HNO_3_ and HF and measured according to DIN EN ISO 11885 / 22036 [42] by ICP-OES (Perkin Elmer, 2100 ZL, USA). After grinding to a fine powder, total N was measured by an elemental analyzer NA 1108 (CE Instruments, Milano, Italy). N/P ratios were calculated based on mass.

### Environmental predictors

LiDAR is a remote sensing technology, which provides structural information on the illuminated surface, including the 3D terrain, vegetation canopy information, and object heights [43]. Point data, including x, y, and z coordinates, can be converted to a digital terrain model and a digital surface model [44]. The laser emits short pulses of light and the sensor records several returns from leaves, branches, and the underlying ground surface [29]. Vegetation heights can be derived from the difference between the ground and the non-ground returns [29]. LiDAR also generates intensity data, reflecting characteristics of objects, which can provide useful information on forest types and tree species [31]. Detailed overviews are provided by Asner et al. [45] and Hyyppä et al. [46].

We used LiDAR point data which has a vertical accuracy of below 10 cm and an average of 4.08 points/m^2^, surveyed by the National Geographic Information Institute (NGII) in South Korea [47]. The point data were pre-processed to identify ground returns, classify all returns, and calculate the normalized vegetation heights. Furthermore, we calculated a set of forest structural predictors using the LAStools software which provides a wide variety of methods to process LiDAR data [48] (Table 1). First, the ground and non-ground points were classified using the lasground module of LAStools. Then, the ground points were used to produce a digital elevation model with the las2dem module, and heights of non-ground points were calculated using the lasheight module. Finally, LiDAR vegetation metrics were derived using the lascanopy module. The maximum height (Hmax) was computed from the maximum point height within a grid cell. Variations of all vegetation point heights within a grid cell were converted to the standard deviation of heights (Hstd), which indicates the structural diversity of the forest. The canopy cover (Hccp) was calculated as the number of LiDAR first returns greater than the cover cutoff (1.37 m by default) divided by the total number of first returns [48]. NDVI was derived from a 4-m Kompsat-2 image obtained on 11th October 2014 [49,50]. We selected the clear-sky image taken at the similar time as the field survey.

**Table 1:** Environmental predictors for digital soil mapping.

	Predictor	Method	Reference
1	Elevation (ELEV)	Las2dem LAStools module	Isenburg [48]
2	Slope degree (SLO)	Slope, aspect, curvature SAGA module	Zevenbergen et al. [53]
3	Catchment area (CA)	Catchment area (Parallel) SAGA module (Multiple flow direction)	Freeman [54]
4	SAGA topographical wetness index (STWI)	SAGA wetness index SAGA module	Böhner et al. [55]
5	Surface curvature (CUR19)	CURV3 program	Park et al. [52]
6	Normalized difference vegetation index (NDVI)	(NIR–Red)/ (NIR+Red)	Tucker and Sellers [56]
7	Maximum height (Hmax)	Lascanopy LAStools module	Isenburg [48]
8	Canopy cover percentage (Hccp)	Lascanopy LAStools module	Isenburg [48]
9	Standard deviation of heights (Hstd)	Lascanopy LAStools module	Isenburg [48]
10	Forest canopy and height (Hch)	Canopy cover percentage (Hccp) x maximum height (Hmax)	-
11	First return intensity average (Hfiravg)	Lasgrid LAStools module	Isenburg [48]

Note: NIR, near-infrared.

Most topographical predictors were calculated with the terrain analysis modules of the open source software SAGA based on the LiDAR DEM [51]. In addition, surface curvature, which reflects the degree of bending of the three-dimensional surface morphology, was calculated with the CURV3 program [52]. To consider the variability of surface configuration, surface curvature values were calculated with different search window sizes of 3 x 3 to 35 x 35 cells. The one with the highest Pearson’s correlation coefficient with the response variables N and P was finally selected as a predictor: 19 x 19 cells (CUR19). All predictors were converted to 10-m cell size via the nearest neighbor resampling method.

### Random forest

Random forest (RF) is an ensemble learning method that operates by building a set of regression trees and averaging the results [57]. Each tree is built using bootstrap samples of the data and a subset of predictors. Providing the number of trees is large, the overall accuracy (out-of-bag error) of the RF converges [57]. Accordingly, the number of trees was set to 1000. The size of the predictor subset (mtry) was tuned by the R package “caret” [58]. The R package "randomForest" [57] was employed as a dependency.

RF is able to model complex nonlinear relationships between soil properties and environmental predictors. It is easier to apply than other supervised learning methods (e.g. neural networks and support vector regression) and does not require much tuning [58–60]. It also has a better interpretability due to the provision of a predictor importance measure. For this measure, the predictor values are permuted. The importance is then determined by the difference in mean square error before and after permutation [59]. Overall, RF has demonstrated good performance in DSM applications [16,61–64].

Predictor selection is reported to influence model performance [65–67]. Recursive feature elimination (RFE), a backward predictor selection method, begins with all predictors and iteratively eliminates the least important predictors one by one based on an initial measure of RF predictor importance until the best predictor remains [58]. At the end, the optimal number of predictors and the final list of selected predictors are returned. The package “caret” provides the functions for RFE [58].

To assess model performance, R^2^ and root mean square error (RMSE) were calculated. For model validation, we used k-fold cross-validation (CV) where the dataset is randomly partitioned into k subsets; one subset is left out for model validation while the remaining subsets are used for model training. The process is repeated k times (once for each fold) and the k estimates of performance are summarized. In k-fold CV, the choice of k determines the size of the test and training dataset. For example, in the case of 10-fold CV, 10% of the data are used for validation and the remaining 90% are used for calibration. The choice of k is usually 5 or 10; however there is no formal rule [58]. Although the subsets are generated randomly, the subdivision still affects model validation results. This can be acknowledged by repetitions of the k-fold CV. Still, the number of repetitions (n) might also affect the estimated model performance; for example, more repetitions lead to better results [68]. We explored 2-, 5-, 10-, 20-fold, and leave-one-out (LOO) CV in n repetitions to account for a total of 100 validation measures: *n* × *k* = 100. Ultimately, 100 R–squares and RMSEs were returned for each soil property. Finally, the cell-wise standard deviation of the corresponding 100 predictions provides an estimate of spatial uncertainty.

## Results

### Descriptive statistics of soil nutrients

Summary statistics for the N and P data are shown in Table 2. The mean N value of the organic layer (N_o_)was higher than that of the A horizon (N_a_). N_o_ had the lowest coefficient of variation (CoV), while total P in the organic layer (P_o_) showed a relatively higher variance based on the standard deviation and CoV. This indicates that the variability in the N/P ratios in the organic layer (N_o_/P_o_) was dependent on P_o_ content, and that there was major P input from the litter fall. The N/P ratio in the A horizon (N_a_/P_a_) showed a higher relative variability than did those in the organic layer, as indicated by the CoV. The mean N_o_/P_o_ was 20.83 ± 4.82 and the mean N_a_/P_a_ was 7.91 ± 2.42.

**Table 2:** Statistical summary of N and P content (mg kg^-1^) and ratios.

	Mean	SD	MIN	Median	MAX	CoV (%)	Skew	Kurt
N_o_	12245	1986	8000	12200	17800	16.22	0.35	2.92
P_o_	624	190	310	610	1240	30.39	0.44	2.97
N_a_	2990	1348	700	2600	7300	45.07	0.81	3.52
P_a_	389	171	160	330	920	43.96	1.40	4.52
N_o_/P_o_	20.83	4.82	12.16	20.17	38.06	23.12	0.76	3.77
N_a_/P_a_	7.91	2.42	1.89	7.78	13.85	30.55	0.21	3.06

Notes: SD, standard deviation; MIN, minimum; MAX, maximum; CoV, coefficient of variation; Skew, skewness; Kurt, kurtosis; N, nitrogen; P, phosphorus; _o_, organic layer; and _a_, A horizon.

### Model validation

**Fig 2:**
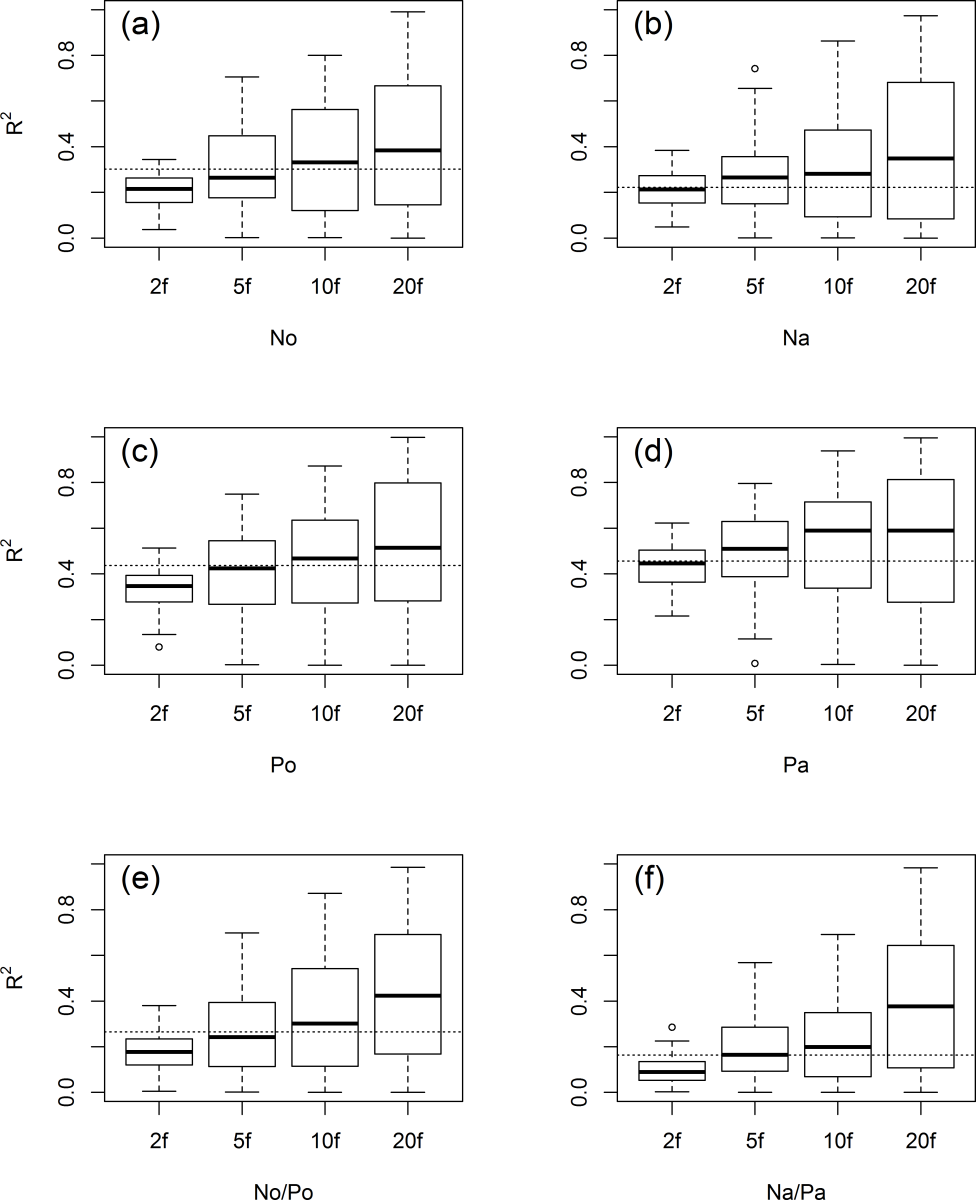
Model validation based on R-square with cross validation methods. The dotted lines indicate the leave-one-out cross-validated result. 2f, 2-fold 50 repetitions; 5f, 5-fold 20 repetitions; 10f, 10-fold 10 repetitions; 20f, 20-fold 5 repetitions; N, nitrogen; P, phosphorus; o, organic layer; and a, A horizon.

**Fig 3:**
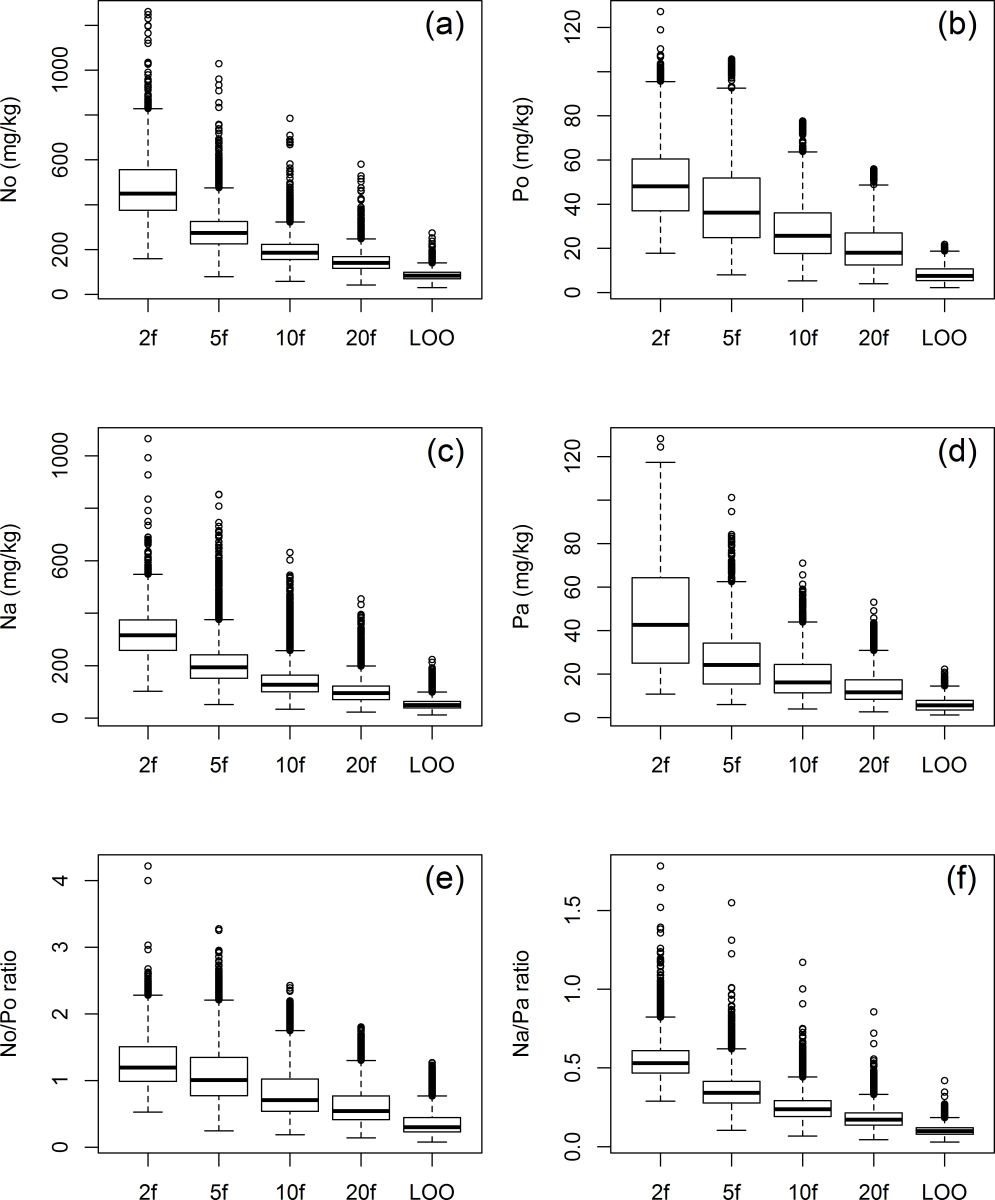
Boxplots showing standard deviations of 100 predicted values for each raster cell with cross validation methods. 2f, 2-fold 50 repetitions; 5f, 5-fold 20 repetitions; 10f, 10-fold 10 repetitions; 20f, 20-fold 5 repetitions; LOO, leave-one-out; N, nitrogen; P, phosphorus; o, organic layer; and a, A horizon.

Fig 2 and S1 Fig show that with increasing k in repeated k-fold CV, mean R-square and RMSE values indicate a better model performance, while R-square and RMSE variance increases as well. Based on mean R-square, the LOO CV results were inferior to the repeated 10-fold and 20-fold, but superior to the repeated 2-fold results. Concerning repeated 5-fold CV, LOO CV was superior for the predictions of the organic layer nutrients, but inferior for the predictions of the mineral soil nutrients. Altogether, mean R-square values were higher for P_o_ and P_a_ compared to N_o_ and N_a_ respectively. The results for N_o_/P_o_ and N_a_/P_a_ were the worst, but showed the highest increase in model performance (mean R-square) with increasing k. Fig 3 shows the standard deviations of all raster cells according to the 100 spatial predictions resulting from the 100 models from the various CV schemes. The mean standard deviation and the variance of the standard deviations decrease with increasing k for all models.

**Fig 4:**
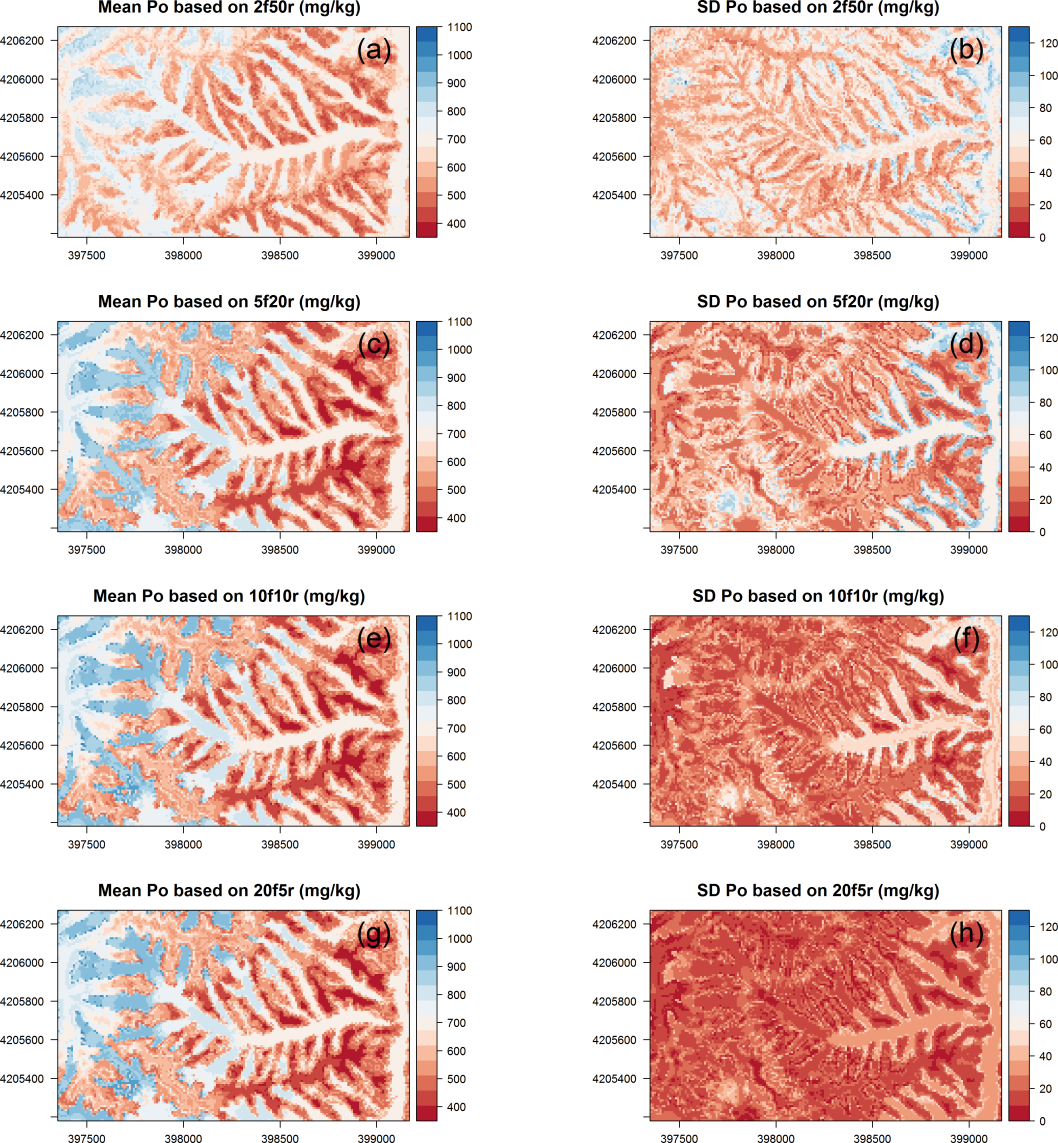
Maps of mean and coefficient of variation (CoV) of 100 models of phosphorus in the organic layer (P_o_) with cross validation methods. 2f50r, 2-fold 50 repetitions; 5f20r, 5-fold 20 repetitions; 10f10r, 10-fold 10 repetitions; 20f5r, 20-fold 5 repetitions.

As an example, spatial prediction patterns of P_o_ including mean values and the standard deviations from the 100 predictions according to the various CV schemes are displayed in Fig 4. In particular, spatial patterns of mean P_o_ of the repeated 5-, 10-, and 20-fold CV are optically very similar (Fig 4C, 4E and 4G). Only the results from repeated 2-fold CV (Fig 4A) show a comparatively smaller range of mean P_o_ values with lower values in the valleys and higher values along ridges. Furthermore, the increase of mean P_o_ values with elevation, which was particularly observable in the concave valley for repeated 5-, 10- and 20-fold CV, is less pronounced for repeated 2-fold CV. As already indicated by Fig 3, standard deviation values decrease with increasing k and a correspondingly bigger calibration dataset. The spatial patterns of the standard deviations show an abrupt increase in the concave valley in the lower part of the study area (Fig 4B, 4D, 4F and 4H).

### Environmental drivers of spatial nutrient patterns

To analyze the influence of topography and vegetation on soil nutrients, the results from repeated 10-fold CV are displayed. These correspond to a comparatively good performance for all soil nutrients based on mean R-square, while R-square variance is not as high as for repeated 20-fold CV (Fig 2). The predictors selected with RFE are shown in Table 3. Surface curvature and elevation were selected for all soil nutrients. For P_o_ and P_a_, they were the only selected predictors. NDVI and LiDAR vegetation predictors (Hfiravg, Hstd, and Hmax) were additionally selected for N_o_. For the N/P ratios parameters corresponding to water flow were additionally selected. While the models for N_0_/P_0_ in correspondence to N_0_ also included vegetation metrics as predictors (Hst, Hmax, and Hch), the model for N_a_/P_a_ included the NDVI instead. We expected that the tree species influenced the spatial pattern of N/P ratios (Fig 1). Tree species were initially also tested as predictors; however, these were not considered important predictors based on previous results. Accordingly, they were excluded due to the simplicity of the model.

**Table 3:** Selected predictors using recursive feature elimination (RFE) based on repeated 10-fold cross validation.

Soil properties	Predictors
N_o_	ELEV, NDVI, Hfiravg, CUR19, STWI, Hstd, Hmax
P_o_	CUR19, ELEV
N_a_	ELEV, CUR19
P_a_	CUR19, ELEV
N_o_/P_o_	CUR19, CA, Hstd, ELEV, Hmax, Hch
N_a_/P_a_	CUR19, CA, NDVI, ELEV, STWI

Notes: ELEV, elevation; CUR19, surface curvature (19 x 19 local window); STWI, SAGA topographical wetness index; CA, Catchment area; SLO, slope degree; NDVI, normalized difference vegetation index; Hfiavg, first return intensity average; Hstd, standard deviations of heights; Hmax, maximum height; Hccp, canopy cover percentage; Hch, forest canopy and height (Hmax X Hccp); N, nitrogen; P, phosphorus; _o_, organic layer; _a_, A horizon.

Our RF model revealed good performance for all soil nutrients based on R^2^ (Fig 2). Mean R-square values ranged from 0.23 to 0.52. P_a_ showed the best result of the validation, while that of the R-square for N_a_/P_a_ was lowest. Models for P showed better results than did models for N.

**Fig 5:**
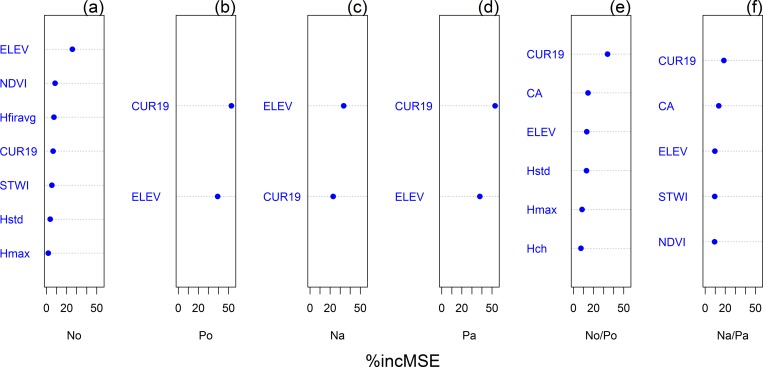
Mean relative importance of predictors for nitrogen and phosphorus based on the increased mean square error (%incMSE) from random forest. N, nitrogen; P, phosphorus; o, organic layer; and a, A horizon.

Fig 5 shows the mean relative predictor importance of the RF models created by repeated 10-fold CV. Terrain predictors exhibited 5.37–53.07% of the reduction in the mean square error (MSE). Surface curvature was the best or second best predictor for all soil nutrients, with the exception of N_o_ (Fig 5); contributed 6.50–53.07% of the MSE. Elevation exhibited a similarly high predictor importance: 9.55–39.22%. NDVI and LiDAR derived vegetation metrics (Hstd, Hmax, Hpdy, and Hfiravg) were also important precitors for the nutrients. The results showing the RF predictor importance were not consistent with the RFE results; however, the two results were similar and there was no difference in the most important predictors (Table 3).

**Fig 6:**
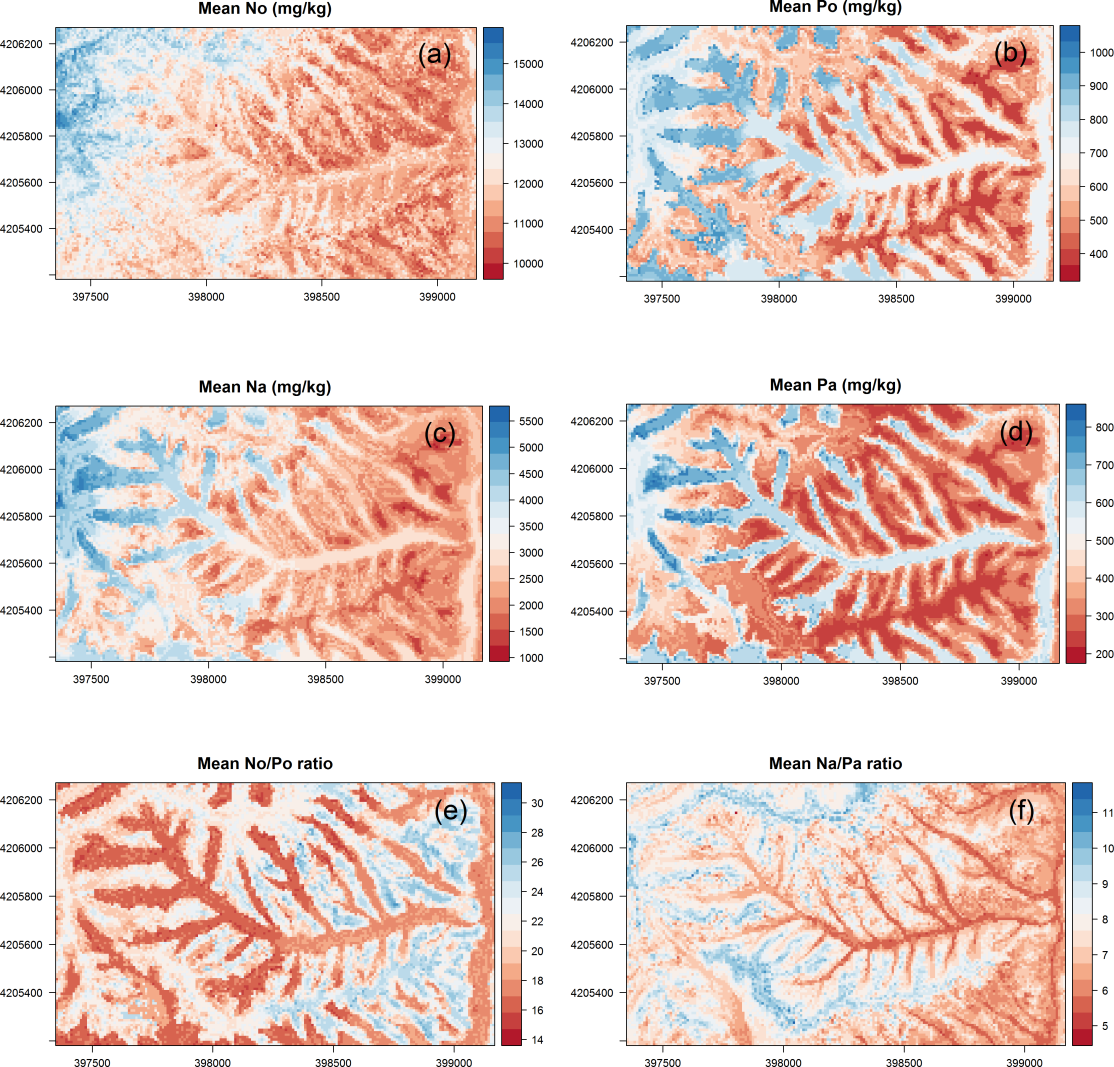
Predicted mean soil N and P content and ratios. N, nitrogen; P, phosphorus; o, organic layer; and a, A horizon.

The map of each nutrient displays the mean of the 100 predictions from repeated 10-fold CV (Fig 6). N_o_ and N_a_ content increased with elevation. We found that P content differed markedly between the upper and lower slopes. N_o_/P_o_ and N_a_/P_a_ were higher on the convex upper slope.

Higher standard deviations of P_o_ and N_o_/P_o_ were found at lower elevations and on the valley floor (S2 Fig). The spatial uncertainties of P_a_ were higher at the upper part of the catchment. Uncertainties of N_o_ (S2 Fig) were similarly complex like the spatial pattern of the mean values (Fig 6A).

## Discussion

### Predictors of soil N and P

In this study, N_o_ (r = 0.58, p<0.001) and N_a_ (r = 0.49, p<0.001) were correlated with elevation. Likewise, Bedison and Johnson [69] also found a strong relationship between N_o_ and elevation (R^2^ = 0.41, P<0.001) in mountainous forested areas in the USA. Additionally, positive relationships between N_a_ and elevation were reported by Kunkel et al. [15], Wang et al. [70] and Peng et al. [13]. The catchment area (CA) and topographical wetness index (TWI) were important predictors of N_o_ in other studies [19,20]. In our study, CA and TWI were not significant for N_o_, whereas N_a_ was correlated with TWI (r = 0.26, p<0.05). According to Aandahl [71], higher nitrogen content is found on the lower slope. Higher N_a_ was found in areas with high elevation and on the lower slope (Fig 6C), which might have higher productivity (plants and microbes) and therefore, higher nitrogen fixation.

Vegetation can determine the spatial distribution of N in forest ecosystems [69,72]. For N_o_, NDVI ranked as the second most important predictor and the LiDAR intensity of first returns (Hfiravg), which is often used as an indicator of forest type [31], was also an important predictor. Although NDVI and LiDAR predictors were not selected as predictors of the N_a_ model, N_a_ was weakly correlated with maximum height (r = 0.24, p<0.05) and standard deviations of heights (r = 0.23, p<0.05). Other studies have found significant relationships between N_a_ and NDVI which can measure vegetation density and aboveground biomass [15,16,73]. This implies that the density of forest cover and forest types affects the N_o_ content and N_o_/P_o_ ratios. Vesterdal et al. [74] reported significant differences for N_o_ but not for N_a_ based on tree species and forest types. However, no relationship was found between P and LiDAR predictors.

As noted, LiDAR-derived predictors are promising for spatial soil predictions. In future studies, vegetation predictors should be applied to forest areas where there is difference in the variation of forest cover. Forest structure (LiDAR metrics) can have an effect on erosion and deposition of materials, which in turn, might alter the soil nutrient content. Hahm et al. [75] confirmed that differences in erosion rates are affected by tree canopy cover. However, to our knowledge, no studies have investigated the relationship between soil erosion, forest structures, and nutrient status using LiDAR data so far.

### Spatial patterns of N/P ratios

We found that N/P ratios increased with surface curvature and were higher on the upper slope compared to the lower slope. This was due to P enrichment of the soil on the lower slope and a more even distribution of N (Fig 6). N_o_/P_o_ and N_a_/P_a_ were strongly related to surface curvature (Fig 6), which implies that P dynamics are affected strongly by topography. This is likely because P was carried from the upper slope by surface and subsurface flows and accumulated on the lower slope, as observed previously in other areas [33]. Soil erosion in the watershed under study is strong due to storm events and steep slopes [76,77]. Consequently, higher soil P content on the lower slope than on the upper slope can lead to higher plant P uptake and higher plant litter P content, leading to a lower N_o_/P_o_. This implies that spatial patterns of N_o_/P_o_ might be generated by the interconnected relationships between soil, topography, and vegetation. Similarly, Uriarte et al. [78] found that soil N/P was correlated with leaf litter N/P, and was determined by topography in a tropical mountainous forest with heavy rainfall and steep slopes.

### Model performance based on different cross validation schemes

We observed the typical bias-variance tradeoff when comparing the various CV schemes as was discussed at length in Hastie et al. [79]. With a higher k, the mean test error decreases, while test error variance increases (Fig 2, S1 Fig). In general, the performance of the learning method varies with the size of the training set. A higher k results in a higher amount of training data, which can be crucial with small datasets. This pattern was consistent with the findings of previous studies. Park and Vlek [80] tested the change in prediction error with different numbers of training soil data sets, and confirmed that the prediction accuracy increases when increasing numbers of soil samples are used for the tuning dataset. A similar decrease in the prediction error was found using various methods for soil prediction according to Ballabio [25]. Generally, 10-fold CV is recommended in most studies [81–86]. Remesan and Mathew [81] noted that the use of very few datasets might result in poorly calibrated models, while high amounts of data for calibration might lead to overfitting. For small sample sizes, model calibration requires all possible datasets to improve the model performance, while validation results can differ markedly depending on which samples are included in the validation [58]. Therefore, Kuhn and Johnson [58] suggested repeated 10-fold CV for small sample sizes because the bias and variance are somewhat balanced and the computational efficiency is good.

The size of the standard deviations of the spatial predictions corresponds to the applied CV scheme (Fig 3). Naturally, a low model bias goes along with low standard deviations. With a high amount of samples included in the training dataset, the training datasets and hence the 100 models are very similar to one another and will, therefore, make similar predictions. That this ensemble of RF models (e.g. from repeated 20-fold or LOO CV) comes along with a high error variance indicates that it is not a good choice, as the corresponding model might be overfitting the data and perform poorly on other data.

## Conclusions

Here, we created the first digital soil maps, showing the spatial pattern of N/P ratios using LiDAR-derived vegetation and topographic predictors. These maps help to identify areas with low nutrient availability. In our study, repeated 10-fold CV was recommended for model validation with small sample sizes. While surface curvature and elevation were mostly sufficient to explain the overall spatial pattern, particularly N contents as well as nutrient ratios in the organic layer benefited from the inclusion of the LiDAR derived vegetation metrics. N/P ratios on the upper slope were higher than those on the lower slope and therefore, productivity on the upper slope might be limited by P in mountainous ecosystems under monsoon conditions. Finally, our analyses show that topographic and vegetation characteristics may help to predict the spatial distribution of nutrients and hence, nutrient limitation in mountainous regions.

## Supporting information

S1 Fig Model validation based on root mean square error (RMSE) with cross validation methods. The dotted lines refer to the leave-one-out cross-validated result. 2f, 2-fold 50 repetitions; 5f, 5-fold 20 repetitions; 10f, 10-fold 10 repetitions; 20f, 20-fold 5 repetitions; N, nitrogen; P, phosphorus; o, organic layer; and a, A horizon.
(TIFF)

S2 Fig Predicted SD nitrogen and phosphorus content and ratios. SD, standard deviation; N, nitrogen; P, phosphorus; o, organic layer; and a, A horizon.
(TIFF)

S1 File Soil nitrogen and phosphorus at soil sampling sites. (XLS)

S2 File Environmental predictors for digital soil mapping. (ZIP)
