# Supplementary figures and images for: Environmental drivers of spatial patterns of topsoil nitrogen and phosphorus under monsoon conditions in a complex terrain of South Korea
Source: PLoS One. 2017 Aug 24;12(8):e0183205. doi: 10.1371/journal.pone.0183205 (PMC5570292; doi:10.1371/journal.pone.0183205)

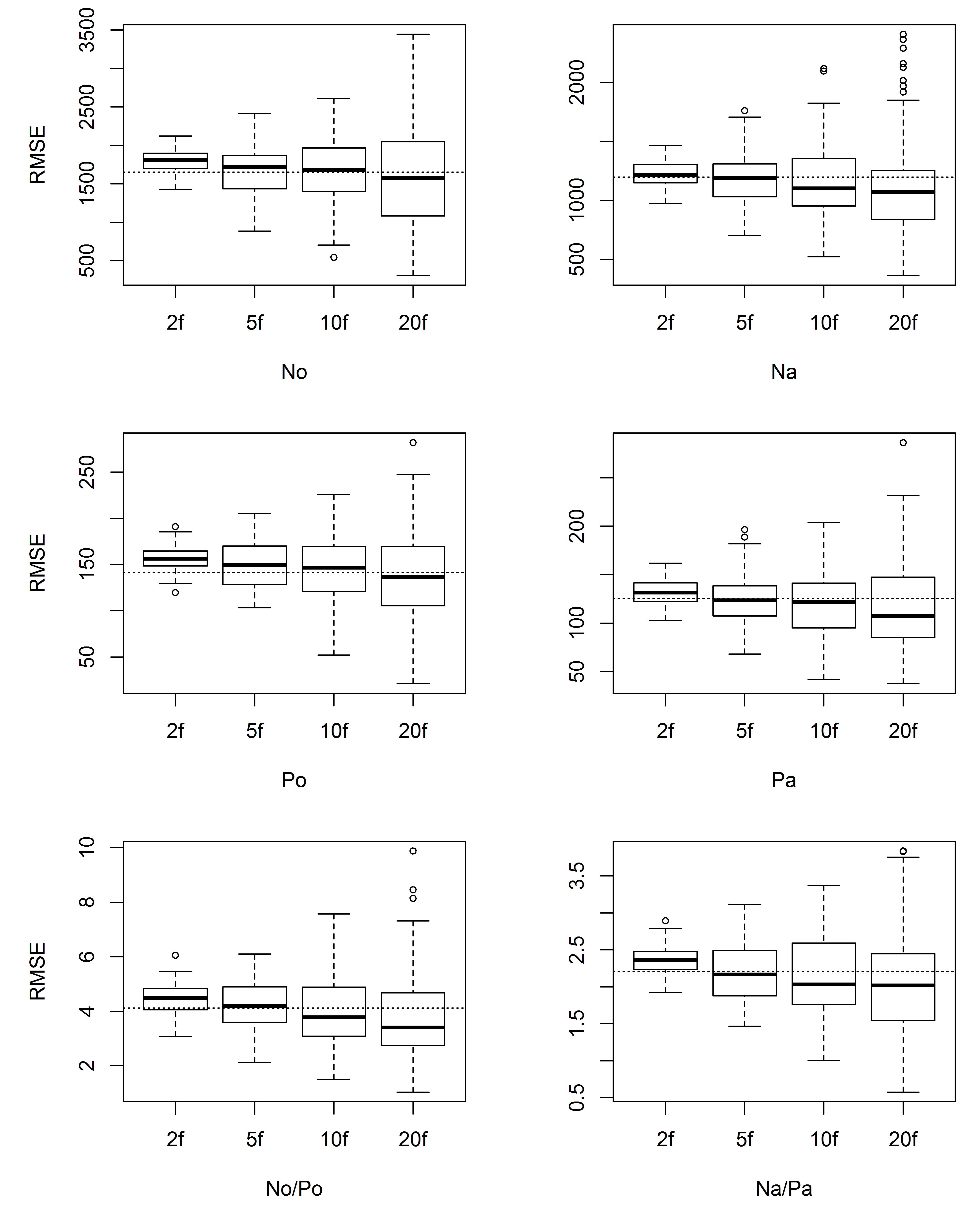

Supplement: S1 Fig — The dotted lines refer to the leave-one-out cross-validated result. 2f, 2-fold 50 repetitions; 5f, 5-fold 20 repetitions; 10f, 10-fold 10 repetitions; 20f, 20-fold 5 repetitions; N, nitrogen; P, phosphorus; o, organic layer; and a, A horizon. (TIFF) [file pone.0183205.s001.tiff]

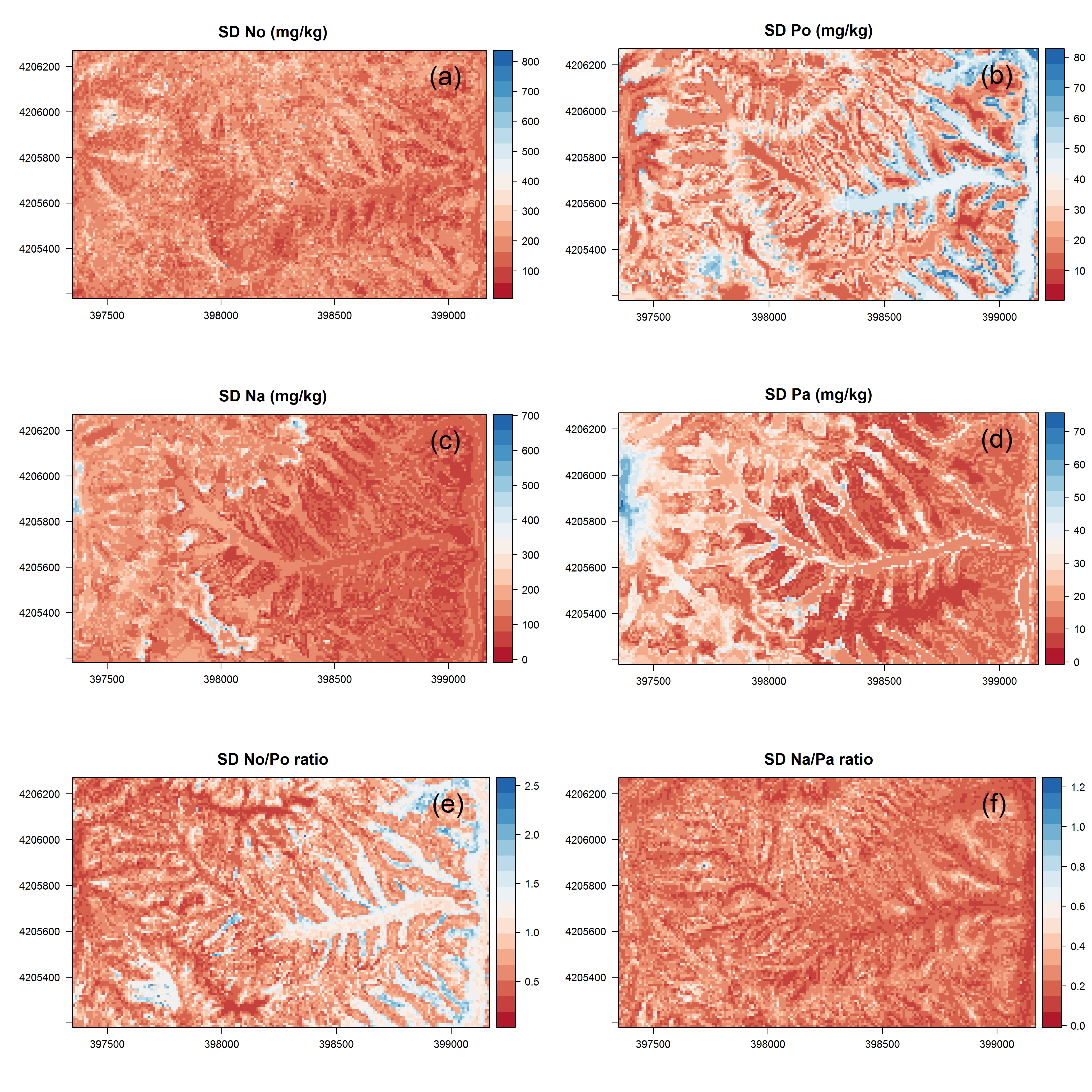

Supplement: S2 Fig — SD, standard deviation; N, nitrogen; P, phosphorus; o, organic layer; and a, A horizon. (TIFF) [file pone.0183205.s002.tiff]
